# Assessment of cerebral venous sinus thrombosis using T_2_^*^-weighted gradient echo magnetic resonance imaging sequences

**Published:** 2016-04-03

**Authors:** Fatemeh Bidar, Fariborz Faeghi, Askar Ghorbani

**Affiliations:** 1 Department of Radiology Technology, School of Allied Medical Sciences, Shahid Beheshti University of Medical Sciences, Tehran, Iran; 2 Iranian Center of Neurological Research, Department of Neurology, Shariati Hospital, Tehran University of Medical Sciences, Tehran, Iran

**Keywords:** Cerebral Venous Sinus Thrombosis, Magnetic Resonance Imaging, Gradient Echo Sequences

## Abstract

**Background:** The purpose of this study is to demonstrate the advantages of gradient echo (GRE) sequences in the detection and characterization of cerebral venous sinus thrombosis compared to conventional magnetic resonance sequences.

**Methods:** A total of 17 patients with cerebral venous thrombosis (CVT) were evaluated using different magnetic resonance imaging (MRI) sequences. The MRI sequences included T_1_-weighted spin echo (SE) imaging, T^*^_2_-weighted turbo SE (TSE), fluid attenuated inversion recovery (FLAIR), T^*^_2_-weighted conventional GRE, and diffusion weighted imaging (DWI). MR venography (MRV) images were obtained as the golden standard.

**Results:** Venous sinus thrombosis was best detectable in T^*^_2_-weighted conventional GRE sequences in all patients except in one case. Venous thrombosis was undetectable in DWI. T^*^_2_-weighted GRE sequences were superior to T^*^_2_-weighted TSE, T_1_-weighted SE, and FLAIR. Enhanced MRV was successful in displaying the location of thrombosis.

**Conclusion:** T^*^_2_-weighted conventional GRE sequences are probably the best method for the assessment of cerebral venous sinus thrombosis. The mentioned method is non-invasive; therefore, it can be employed in the clinical evaluation of cerebral venous sinus thrombosis.

## Introduction

Cerebral venous thrombosis (CVT) is a condition that affects about five people per million and accounts for 0.5% of all strokes. It results from thrombosis of the intracranial veins and dural venous sinuses that drain blood from the brain.^1^

CVT is a neurological condition with nonspecific symptoms. Magnetic resonance imaging (MRI) in conjunction with MR venography (MRV) is the most sensitive technique for the diagnosis of CVT.^2^ The important finding in CVT on MRI images is the absence of normal flow void on T_1_ and T_2_-weighted images (WIs). Signal changes depend on the age of the thrombosis and the amount of residual flow. Common manifestations include headache, focal neurologic deficits, seizures, and variable consciousness. Other signs worth mentioning are obscuration of vision, nausea, papilledema, cranial nerve palsies, and coma.^3^ Most patients with deep venous sinus thrombosis show signs of increased intracranial pressure.^4^ Isolated subarachnoid hemorrhage may also occur rarely due to CVT.^5^ Conventional T_1_ and T_2_-weighted spin echo (SE) sequences are not sensitive enough to exhibit CVT particularly in acute phases.^1^ Theoretically, T^*^_2_-weighted gradient recalled echo (GRE) sequences can be more efficient in the depiction of CVT due to their higher sensitivity to magnetic susceptibility differences. In this study, we employed various MRI sequences to evaluate patients with CVT.

## Materials and Methods

In this study, 17 patients with CVT were examined. All the patients had undergone MRI studies. MRI images were obtained using a 1.5 T MR scanner (MAGNETOM Avanto, Siemens) equipped with 18 receiver channels. The imaging sequences included T_1_-WI [repetition time/echo time (TR/TE), 430/9 ms], T_2_-WI [(TR/TE), 3720/100 ms], fluid attenuated inversion recovery (FLAIR) [TR/TE/inversion time (TI), 7100/92/2232.4 ms], diffusion WI (DWI) [(TR/TE), 720/126 ms, flip angle (FA); 90°, matrix size; 192 × 192 and field of view (FOV); 230 × 230 mm).

T^*^_2_-weighted conventional GRE images were obtained from all subjects (TR-720 ms; TE-26 ms; FA = 20°, axial planes; slice thickness, 5 mm; matrix size, 256 × 110; and FOV, 138 × 270 mm).

Afterward, three-dimensional enhanced MRV images [(TR/TE), 3/1 ms, FA = 25°] were acquired during injection of Dotarem. A dose of 0.1 mmol of Dotarem was injected at the rate of 2 ml/s. All MRI images were reported by two experienced radiologists and a neurologist. The reviewers were in strong agreement regarding the visualization of thrombosis on various evaluated imaging sequences. However, there were two conflicts in the detection of thrombosis on DWI sequences between the radiologists. One of the radiologists believed that the thrombosis was detectable in the two mentioned DWI images, while the other radiologist and the neurologist believed that the thrombosis was invisible in those cases. The final diagnosis was that the thrombosis was undetectable (not seen). Signal changes were evaluated relative to the gray matter for each pulse sequence. The data were entered into SPSS software (version 16, SPSS Inc., Chicago, IL, USA) and the following formulas were employed to evaluate confidence interval (CI) and intraclass correlation coefficient (ICC).

CI = Sensitivity ± 1.96 * SE


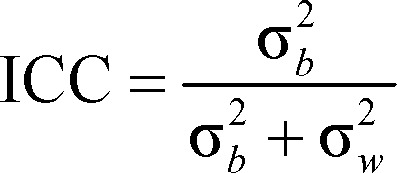


Ơ ^2^_w_ is the pooled variance within subjects, and Ơ ^2^_b_ is the variance of the trait between the subjects.

Visualization of venous sinus thrombosis was assessed based on the following criteria:

1. Visible (1-1 high signal, 1-2 low signal)
2. Iso signal (insufficient for diagnosis)
3. Not seen.

## Results

A total of 17 patients (75.5% female) with CVT were involved in this study with the mean age of 45 ± 18.76 ranging from 4 to 72 years (Table 1). Sensitivity tests were employed to evaluate the diagnostic value of T^*^_2_-weighted GRE, FLAIR, T_2_-weighted, T_1_-weighted, and DWI sequences. Sensitivity value of T^*^_2_-weighted GRE, FLAIR, T_2_-weighted, T_1_-weighted and DWI methods equaled 0.94, 0.82, 0.52, 0.64, 0.06, respectively. CI of the mentioned sequences was (0.85, 0.99), (0.69, 0.92), (0.38, 0.59), (0.53, 0.77), and (0.00, 0.09), respectively. Sensitivity value of T^*^_2_-weighted GRE, FLAIR, T_2_-weighted, T_1_-weighted and DWI methods for subacute cases equaled 0.9, 1, 0.55, 1, 0.09 and for acute cases equaled 1, 0.5, 0.5, 0.0 CI of the mentioned sequences for subacute cases was (0.57, 0.99), (0.77, 1), (0.25, 0.82), (0.76, 1), (0.04, 0.42) and for acute cases was (0.75, 1), (0.14, 0.86), (0.14, 0.86), (0.0, 0.048), (0.0, 0.48). ICC test showed significant agreement between all methods in the diagnosis of CVT (P < 0.05). ICC with the exact value of 0.27 indicates a weak agreement among the evaluated sequences.

**Figure 1:**
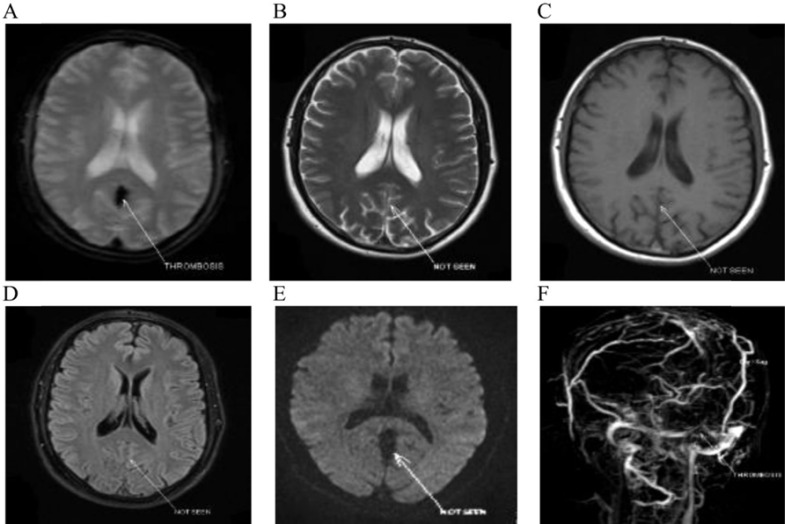
Cerebral venous thrombosis in a 50-year-old male with history of headache

T^*^_2_-weighted conventional GRE sequences were superior in the detection of CVT in comparison with other techniques (Figure 1, A-F).

## Discussion

CVT is a cerebrovascular disorder that most often affects young adults and children.^6^ Contrast enhanced MRV (CE-MRV) can perfectly demonstrate the thrombosis, small vein details, and collaterals.^7^ Conventional MRI is unreliable. It is sometimes difficult to decide whether the signal within a cerebral vein corresponds to the flow or to the thrombosis. An acute clot (up to 5 days) is isointense to the gray matter on T_1_-WI (and therefore easily missed) and hypointense on T_2_-WI.^8^

In this study, areas of thrombosis were more prominently shown on GRE pulse sequence due to the high sensitivity of this sequence to tissue susceptibility differences. Other techniques were not as beneficial as T^*^_2_--weighted GRE sequences. Previous investigations have evaluated T^*^_2_--weighted GRE sequences in the diagnosis of CVT. 

**Table 1:** Visual assessment of cerebral venous sinus thrombosis in various sequences

**Sex/age ** **(year)**	**Location of ** **thrombosis**	**T**_1_	**T**_2_	**FLAIR**	**DWI**	T^*^_2_**GRE**	**Stage**	**Symptoms**
M/50	SSS + Straight sinus	1-1	1-1	1-1	3	1-2	S	Headache, nausea
F/4	SS	1-1	1-1	1-1	3	1-2	S	Convulsion, variable consciousness
F/61	TS	1-1	3	1-1	3	1-2	S	Headache, cerebral palsy
F/26	SS + TS	3	3	3	3	1-2	A	Headache, nausea
F/53	TS + SS	1-1	3	1-1	3	1-2	S	Headache, obscuration of vision, nausea
F/38	TS + SS	1-1	3	1-1	3	1-2	S	Headache, epilepsy
F/57	TS	1-1	2	1-1	3	1-2	S	Headache, nausea, seizure
F/30	SSS + TS + SS	1-1	2	1-1	3	1-2	S	Headache, seizure, nausea
F/64	TS	3	3	1-1	3	1-2	A	Headache, variable consciousness, nausea
F/49	TS	2	1-1	2	3	1-2	A	Headache, cerebral palsy, seizure
F/27	SS	1-1	1-1	1-1	3	1-2	S	Headache, nausea, convulsion
M/72	TS + SS	1-1	1-1	1-1	3	3	S	Headache, epilepsy, obscuration of vision
M/26	TS	3	3	1-1	3	1-2	A	Headache, nausea
F/48	SSS + TS	1-1	1-1	1-1	1-1	1-2	S	Headache, nausea
F/41	TS	2	1-1	2	3	1-2	A	Headache, convulsion
M/55	TS + SS	1-1	1-1	1-1	3	1-2	S	Headache, variable consciousness
F/71	SSS	2	1-1	1-1	3	1-2	A	Headache, nausea, convulsion

M: Male; F: Female; TS: Transverse sinus; SS: Sigmoid sinus; SSS: Superior sagittal sinus; GRE: Gradient echo; FLAIR: Fluid attenuated inversion recovery; DWI: Diffusion weighted imaging

1: Visible (1-1 high signal, 1-2 low signal), 2: Iso signal (insufficient for diagnosis), 3: Not seen, A: Acute (< 5 days), S: Sub-acute (5-15 days)

Note the hypointense area on $T_{2}^{*}$-weighted gradient echo sequence (arrow) (A), thrombosis is invisible on T_2_-weighted image (B), T_1_-weighted image (C), fluid attenuated inversion recovery (D), diffusion weighted imaging (E), the absence of flow signal on contrast-enhanced magnetic resonance venography image represents the precise location of thrombosis (F)

Ihn et al.^9^ have assessed the efficacy of T^*^_2_-weighted GRE in 11 patients with CVT and compared the results with other conventional sequences. They reported that T^*^_2_-weighted GRE was able to visualize marked signal loss from CVT in all the studied patients.

Fellner et al.^10^ have evaluated the importance of T^*^_2_-weighted GRE MRI in the diagnosis of cortical vein thrombosis in six cases of CVT. In four cases, which involved superficial venous thrombosis, the susceptibility effect on GRE sequences in the thrombosed cortical veins was the most pronounced finding compared with those seen on other pulse sequences.

We found that conventional sequences were not satisfying in the detection of acute stage. CE-MRV can perfectly demonstrate the thrombus, small vein details, and collaterals. CE-MRV showed the extension and the exact location of thrombosis. SE-MRV revealed the clot in all of the assessed patients. It can be concluded that T^*^_2_-weighted GRE sequences are more sensitive to paramagnetic effects compared with the sequences based on SE. T^*^_2_-weighted GRE sequences are effective in the detection of CVT, particularly in acute stage.

## Conclusion

These sequences can be used as a standard MR pulse sequence. MRV is impressively helpful in the diagnosis and the determination of the extent of CVT.

## Notes:

**How to cite this article:** Bidar F, Faeghi F, Ghorbani A. Assessment of cerebral venous sinus thrombosis using T_2_^*^-weighted gradient echo magnetic resonance imaging sequences. Iran J Neurol 2016; 15(2): 96-9.

## References

[1] Haacke EM, Reichenbach JR (2011). Susceptibility weighted imaging in MRI: basic concepts and clinical applications.

[2] Lafitte F, Boukobza M, Guichard JP, Hoeffel C, Reizine D, Ille O (1997). MRI and MRA for diagnosis and follow-up of Cerebral Venous Thrombosis (CVT). Clin Radiol.

[3] Rodallec MH, Krainik A, Feydy A, Helias A, Colombani JM, Julles MC (2006). Cerebral venous thrombosis and multidetector CT angiography: tips and tricks. Radiographics.

[4] Nabil Sheri M, Kashif A (2012). Chronic deep venous thrombosis of the petrosal sinus causing multiple cranial nerve palsy. Iran J Neurol.

[5] Ghandehari K, Riasi HR, Noureddine A, Masoudinezhad S, Yazdani S, Mirzae MM (2013). Safety assessment of anticoagulation therapy in patients with hemorrhagic cerebral venous thrombosis. Iran J Neurol.

[6] Stam J (2005). Thrombosis of the cerebral veins and sinuses. N Engl J Med.

[7] Huston J, Ehman RL (1993). Comparison of time-of-flight and phase-contrast MR neuroangiographic techniques. Radiographics.

[8] Dormont D, Anxionnat R, Evrard S, Louaille C, Chiras J, Marsault C (1994). MRI in cerebral venous thrombosis. J Neuroradiol.

[9] Ihn YK, Jung WS, Hwang SS (2013). The value of T2*-weighted gradient-echo MRI for the diagnosis of cerebral venous sinus thrombosis. Clin Imaging.

[10] Fellner FA, Fellner C, Aichner FT, Molzer G (2005). Importance of T2*-weighted gradient-echo MRI for diagnosis of cortical vein thrombosis. Eur J Radiol.

